# Clinical Characteristics and Outcomes of Patients with High Ankle-Brachial Index from the IMPACT-ABI Study

**DOI:** 10.1371/journal.pone.0167150

**Published:** 2016-11-23

**Authors:** Hitoshi Nishimura, Takashi Miura, Masatoshi Minamisawa, Yasushi Ueki, Naoyuki Abe, Naoto Hashizume, Tomoaki Mochidome, Mikiko Harada, Kunihiko Shimizu, Wataru Shoin, Koji Yoshie, Yasutaka Oguchi, Soichiro Ebisawa, Hirohiko Motoki, Atsushi Izawa, Jun Koyama, Uichi Ikeda, Koichiro Kuwahara

**Affiliations:** Department of Cardiovascular Medicine, Shinshu University School of Medicine, Matsumoto, Japan; Osaka University Graduate School of Medicine, JAPAN

## Abstract

**Background:**

Reduced ankle–brachial index (ABI) is a predictor of cardiovascular events. However, the significance of high ABI remains poorly understood. This study aimed to assess the characteristics and outcomes of patients with high ABI.

**Methods:**

The IMPACT-ABI study was a retrospective cohort study that enrolled and examined ABI in 3,131 patients hospitalized for cardiovascular disease between January 2005 and December 2012. From this cohort, 2,419 patients were identified and stratified into two groups: high ABI (> 1.4; 2.6%) and normal ABI (1.0–1.4; 97.3%). The primary endpoint was the cumulative incidence of major adverse cardiovascular events (MACE), including cardiovascular-associated death, myocardial infarction, and stroke.

**Results:**

Compared with the normal ABI group, patients in the high ABI group showed significantly lower body mass index (BMI) and hemoglobin level, but had higher incidence of chronic kidney disease and hemodialysis. Multivariate logistic regression analysis revealed that hemodialysis was the strongest predictor of high ABI (odds ratio, 6.18; 95% confidence interval (CI), 3.05–12.52; P < 0.001). During the follow-up (median, 4.7 years), 172 cases of MACE occurred. Cumulative MACE incidence in patients with high ABI was significantly increased compared to that in those with normal ABI (32.5% vs. 14.5%; P = 0.005). In traditional cardiovascular risk factors-adjusted multivariate Cox proportional hazard analysis, high ABI was an independent predictor of MACE (hazard ratio, 2.07; 95% CI, 1.02–4.20; P = 0.044).

**Conclusion:**

Lower BMI, chronic kidney disease, and hemodialysis are more frequent in patients with high ABI. Hemodialysis is the strongest predictor of high ABI. High ABI is a parameter that independently predicts MACE.

## Introduction

Peripheral artery disease (PAD) is a well-established predictor of all-cause and cardiovascular mortality and morbidity [1, 2]. The ankle-brachial index (ABI) is a simple and useful method that provides information on the presence of PAD [3]. According to the latest ACCF/AHA guidelines for the management of patients with PAD, ABI values between 1.00 and 1.40 are within the normal range, and values > 1.4 indicate non-compressible arteries [4]. High ABI values typically indicate arterial medial calcification, which is very common in patients with diabetes mellitus and end-stage chronic kidney disease [5, 6].

While the majority of previous studies have focused on patients with low ABI and excluded patients with high ABI, a few studies have evaluated the baseline characteristics and outcomes in patients with high ABI. Aboyans et al. reported a strong association between diabetes mellitus and high ABI [7]. On the contrary, Wattanakit et al. demonstrated that individuals with high ABI values were not characterized by a more adverse atherosclerosis risk factor profile, including diabetes mellitus [8]. Therefore, the association between elevated ABI values and prevalent disease remains controversial.

Previous studies have reported the association of high ABI and cardiovascular mortality and morbidity. In the Strong Heart Study (SHS), which was a large-scale prospective study, high ABI was strongly associated with total and cardiovascular mortality [9]. However, high ABI was not associated with cardiovascular mortality and cardiovascular events in multivariate analysis in the Cardiovascular Health Study (CHS) [10]. As stated, the baseline characteristics and prognosis of patients with high ABI compared with those with normal ABI values remain largely unknown.

Therefore, the purpose of this study was to evaluate (1) the characteristics of patients with high ABI and (2) the association between a high ABI value and future cardiovascular events and mortality.

## Methods

### Study design

The current study was performed using integrated data from the impressive predictive value of ABI for clinical long-term outcomes in patients with cardiovascular disease examined by ABI (IMPACT-ABI) study. The IMPACT-ABI study was a retrospective cohort study that enrolled 3,131 consecutive patients who were admitted to Shinshu University for cardiovascular disease and examined by ABI between January 2005 and December 2012. Clinical, demographic, laboratory, and follow-up data were collated from hospital records or by contacting patients and their family. The present study was registered with the University Hospital Medical Information Network Clinical Trials Registry (UMIN-CTR), as accepted by the International Committee of Medical Journal Editors (UMIN-ID; 000020276). The study protocol was performed in accordance with the ethical guidelines of the Declaration of Helsinki and was approved by the Ethics Committee of Shinshu University School of Medicine. Because of the retrospective nature of the current study, informed written consent for participation was not obtained from patients and data were analyzed anonymously.

**Fig 1:**
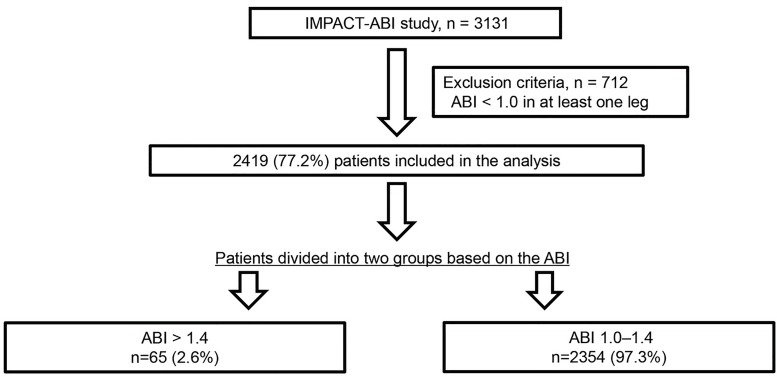
Study flow diagram illustrating the inclusion process and exclusion criteria. Patients were stratified into two groups based on their ankle-brachial index (ABI) values.

Among the potential subjects, 712 patients were excluded due to ABI values < 1.0 in at least one leg, and 2,419 patients were subsequently enrolled. The study cohort was divided into two groups based on ABI: normal ABI group and high ABI group. Based on the latest ACCF/AHA guidelines [4], the normal ABI group participants had ABI values of 1.0–1.4 in both legs. The high ABI group had ABI values of > 1.4 in both legs, or > 1.4 in one leg and 1.0–1.4 in the other (Fig 1).

### ABI measurement

For ABI measurement, systolic blood pressure in the upper extremities (both brachial arteries) and lower extremities (both dorsalis pedis and posterior tibial arteries) were measured using the Form PWV/ABI (Omron Colin, Tokyo, Japan) with the patient in the supine position for at least 10 minutes of bed rest. This instrument is an automated device that can simultaneously measure blood pressure in the bilateral upper and lower extremities via the oscillometric method. ABI was calculated by dividing the systolic blood pressure in the lower extremity by the highest of the bilateral systolic blood pressure values in the upper extremity.

### Study definitions

All-cause death was defined as any death recorded during the follow-up period. Cardiovascular-associated death was defined as mortality due to acute myocardial infarction, significant cardiac arrhythmia, congestive heart failure, stroke, or other cardiovascular causes [11]. Myocardial infarction was defined as a 2-fold rise in serum troponin I or in creatine phosphokinase (CK)-MB isoenzyme to at least twice the upper normal limits, associated with acute onset of prolonged typical ischemic chest pain, ST-segment elevation of at least 1 mm in two contiguous ECG leads, or ST-segment depression of at least 0.5 mm in two contiguous leads [11]. Stroke was defined as ischemic stroke that persisted for ≥ 24 hours or evidence of infarction on magnetic resonance imaging [11].

Diabetes mellitus was defined as fasting blood glucose levels ≥ 7 mmol/l and/or a casual plasma glucose levels ≥ 11.1 mmol/l, HbA1c ≥ 6.5%, or by clinical use of insulin or hypoglycemic agents. Hypertension was defined as systolic blood pressure ≥ 140 mmHg and/or diastolic blood pressure ≥ 90 mmHg, or current treatment with antihypertensive agents. Dyslipidemia was defined as total cholesterol ≥ 5.7 mmol/l; or alternatively, low-density lipoprotein cholesterol levels ≥ 3.6 mmol/l, high-density lipoprotein cholesterol levels ≤ 1.0 mmol/l, triglyceride levels ≥ 1.7 mmol/l, or current treatment with cholesterol-lowering agents. Coronary artery disease was defined as a history of angina and/or previous myocardial infarction. Previous heart failure was defined as a prior heart failure diagnosis according to the Framingham criteria [12] or current treatment for heart failure. Smoking was defined as current smoking status. Body mass index (BMI) was calculated by dividing the participant’s weight (kg) by the square of their height (m). Estimated glomerular filtration rate (eGFR) was estimated using the Japanese equation to estimate kidney function as follows: eGFR (ml/min/1.73 m^2^) = 194×serum creatinine^−1.094^×age^−0.287^ for male patients, and the same formula multiplied by 0.739 was used for female patients [13]. Chronic kidney disease was defined by an eGFR < 60 ml/min/1.73 m^2^ [14].

### Endpoint

The primary endpoint of this study was composite of major adverse cardiovascular events (MACE), including cardiovascular-associated death, myocardial infarction, and stroke. The secondary end-point was all-cause death, and cardiovascular-associated death.

### Statistical methods

All statistical analyses were performed using SPSS version 22 software (SPSS Ink., Chicago, IL, USA). Normal data distribution was assessed via the Shapiro-Wilk test. Continuous variables were reported as median and interquartile range (25^th^–75^th^ percentile) for variables that were not normally distributed. Categorical variables were reported as numbers and percentages. Patient characteristics among each group were compared by the Chi-squared test for categorical variables and the Mann-Whitney U test for continuous variables. Overall occurrences of MACE, all-cause death, and cardiovascular-associated death were calculated using the Kaplan-Meier method, and differences between the groups were compared by the log-rank test. Associations of variables, including traditional cardiovascular risk factors [15], with high ABI were calculated via univariate logistic regression analyses. Variables that exhibited a P-value of < 0.05 in the univariate analyses were included into the multiple logistic regression models. Adjusted odd ratios (OR) and 95% confidence interval (CI) for high ABI were obtained by multiple logistic regression analysis. Univariate Cox proportional hazard analyses were performed to identify the independent predictors of MACE. Variables that exhibited P-value of < 0.05 in the univariate analysis were included in the multivariate model. The magnitude of the relationship between the variables and MACE was expressed as the hazard ratio (HR) and 95% CI. P-values of < 0.05 were considered statistically significant.

## Results

### Baseline characteristics

Baseline characteristics are summarized in Table 1. A total of 2,419 participants were examined in the current study. The median age was 68 years and the majority of patients were male (70.7%). Causes of admission among participants included acute myocardial infarction (4.0%), unstable angina pectoris (1.4%), stable angina pectoris (26.4%), other atherosclerotic diseases (26.9%), arrhythmia (21.9%), heart failure (15.3%), and other ailments (4.1%). Based on ABI, all patients were divided into two groups: high ABI group (n = 65; 2.6%) and normal ABI group (n = 2,354; 97.3%) according to high and normal ABI values, respectively. No significant differences were found in age, sex, hypertension, diabetes mellitus, smoking status, atrial fibrillation, and previous coronary heart disease, cerebral infarction, and heart failure between each group. However, high ABI values were significantly associated with lower BMI, hemoglobin and eGFR, chronic kidney disease and more frequent hemodialysis treatment, as compared with the normal ABI values. Notably, fewer patients exhibited dyslipidemia and previous myocardial infarction in the high ABI group than in the normal ABI group.

**Table 1:** Baseline characteristics.

Variables	Overall	ABI > 1.4	ABI 1.0–1.4	P value
	n = 2419	n = 65	n = 2354	
Age	68 (59–76)	68 (59–76)	67 (57–74)	0.593
female (%)	710 (29.3%)	14 (21.5%)	696 (29.5%)	0.161
ABI	1.13 (1.08–1.19)	1.34 (1.26–1.45)	1.13 (1.08–1.18)	< 0.001
BMI (kg/m^2^*)*	23.3 (21.1–25.8)	21.8 (18.8–25.0)	23.3 (21.1–25.8)	0.003
Hypertension (%)	1,392 (57.5%)	36 (55.3%)	1,356 (57.6%)	0.721
Dyslipidemia (%)	1,031 (42.6%)	19 (29.2%)	1,012 (42.9%)	0.023
Diabetes (%)	609 (25.1%)	18 (27.6%)	591 (25.1%)	0.636
Smoking habit (%)	1,042 (43.0%)	23 (35.3%)	1,019 (43.2%)	0.195
eGFR (ml / min / 1.73 m^2^)	66.4 (53.4–79.1)	59.9 (21.1–80.9)	66.5 (53.7–79.1)	0.021
Hemodialysis (%)	81 (3.3%)	13 (20.0%)	68 (2.8%)	< 0.001
Chronic kidney disease (%)	853 (35.2%)	32 (49.2%)	821 (34.8%)	0.017
Atrial fibrillation (%)	297 (12.2%)	9 (13.8%)	288 (12.2%)	0.701
Coronary heart disease (%)	411 (16.9%)	6 (9.2%)	405 (17.2%)	0.091
Previous MI (%)	336 (13.8%)	3 (4.6%)	333 (14.1%)	0.028
Previous CI (%)	138 (5.7%)	7 (10.7%)	131 (5.5%)	0.075
Previous heart failure (%)	163 (6.7%)	4 (6.1%)	159 (6.7%)	0.848
Hb (g/L)	140 (128–151)	132 (114–147)	140 (128–152)	0.001
Medication				
Aspirin (%)	1,005 (41.5%)	20 (30.7%)	985 (41.8%)	0.035
Thienopyridines (%)	543 (22.4%)	11 (16.9%)	532 (22.5%)	0.202
Cilostazol (%)	65 (2.6%)	2 (3.0%)	63 (2.6%)	0.898
ACEI / ARB (%)	1,091 (45.1%)	28 (43.0%)	1,063 (45.1%)	0.008
Beta-blockers (%)	647 (26.7%)	18 (27.6%)	629 (26.7%)	0.947
Statin (%)	916 (37.8%)	10 (15.3%)	906 (38.4%)	< 0.001
Insulin (%)	83 (3.4%)	2 (3.0%)	81 (3.4%)	0.873
Warfarin (%)	473 (19.5%)	15 (23.0%)	458 (19.4%)	0.607

Data are shown as median (interquartile range), or n (%). Abbreviations: ABI, ankle brachial index; ACEI, angiotensin converting enzyme inhibitor; ARB, angiotensin receptor blockers; BMI, body mass index; CI, cerebral infarction; eGFR, estimated glomerular filtration rate; Hb, hemoglobin; MI, myocardial infarction.

Prescription rates differed between the groups. The use of angiotensin-converting enzyme inhibitors and/or angiotensin receptor blockers, statins, and aspirin in patients with high ABI values was significantly reduced compared with that in those with normal ABI values.

### Predictors of high ABI

Univariate and multivariate logistic regression analyses adjusted for traditional cardiovascular risk factors were performed to assess the power of the variables on high ABI (Table 2). A multivariate logistic regression model indicated that hemodialysis treatment was the strongest predictor of high ABI (OR, 6.18; 95% CI, 3.05–12.52; P < 0.001). Lower BMI was also associated with high ABI (OR, 0.93; 95% CI, 0.87–0.99; P = 0.049).

**Table 2:** Univariate and multivariate logistic regression analyses for high ankle brachial index.

	Univariate analysis		Multivariate analysis	
Variables	OR (95% CI)	P value	OR (95% CI)	P value
Age (for each 1–year increase)	0.99 (0.97–1.01)	0.651		
female	0.65 (0.36–1.18)	0.164		
BMI (for each 1–kg/m^2^ increase)	0.91 (086–0.97)	0.004	0.93 (0.87–0.99)	0.049
Coronary heart disease	0.48 (0.21–1.14)	0.098		
Previous myocardial infarction	0.29 (0.09–0.94)	0.039	0.36 (0.11–1.17)	0.091
Previous cerebral infarction	2.04 (0.91–4.57)	0.081		
Hypertension	0.91 (0.55–1.50)	0.721		
Dyslipidemia	0.53 (0.31–0.92)	0.025	0.74 (0.42–1.31)	0.307
Diabetes	1.14 (0.65–1.98)	0.636		
Atrial fibrillation	1.15 (0.56–2.35)	0.701		
Hemodialysis	8.40 (4.37–16.1)	< 0.001	6.18 (3.05–12.52)	<0.001
Smoker	0.71 (0.42–1.19)	0.197		
Hb (for each 0.1 g/L increase)	0.80 (0.70–0.91)	0.001	0.89 (0.78–1.02)	0.897
Previous heart failure	0.90 (0.32–2.52)	0.848		

BMI, body mass index; CI, confidence interval; Hb, hemoglobin; OR, odds ratio.

### Incidence of MACE, all-cause death, and cardiovascular-associated death

The median follow-up period was 4.7 years (range, 2.9–7.2). During the follow-up period were registered 172 MACE cases, including 115 cardiovascular-associated deaths, 58 strokes, and 27 myocardial infarctions. A total of 302 deaths (cardiovascular, 115; cancer, 75; respiratory failure, 30; infection, 6; renal failure, 7; decrepitude, 11; and other, 58) occurred.

**Fig 2:**
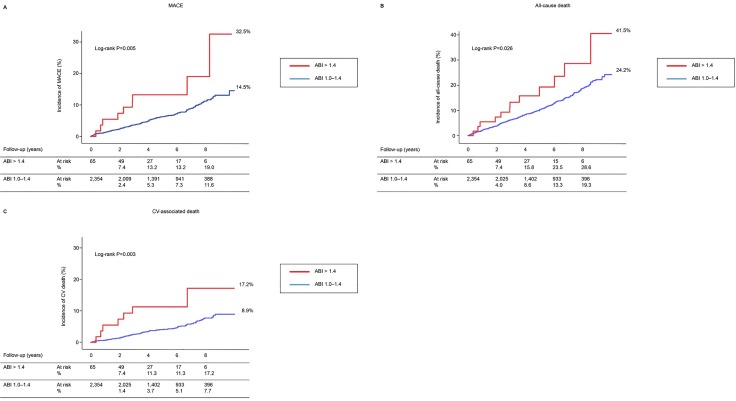
Kaplan-Meier analysis for (A) major adverse cardiovascular (CV) events (MACE) (CV-associated death, myocardial infarction, and stroke), (B) all-cause death, (C) CV-associated death, according to the ankle brachial index (ABI) categories.

In patients with high ABI, a total of 9 MACE (cardiovascular-associated death, 7; stroke, 1; myocardial infarction, 1) and 12 all-cause deaths occurred. In patients with normal ABI, a total of 163 MACE (cardiovascular-associated death, 108; stroke, 57; myocardial infarction, 26) and 290 all-cause deaths occurred. Kaplan-Meier analysis demonstrated that MACE incidence was significantly higher in patients with high ABI values than in those with normal ABI values (32.5% vs. 14.5%; log rank P < 0.005) (Fig 2A). Similarly, the cumulative incidences for all-cause and cardiovascular-associated deaths were significantly higher in patients with high ABI than in those with normal ABI (41.5% vs. 24.2%, log rank P = 0.026; 17.2% vs. 8.9%, log rank P = 0.003, respectively) (Fig 2B and 2C).

### Predictors of MACE, all-cause death, and cardiovascular-associated death

Cox proportional hazard analyses were performed to evaluate the prognostic value of ABI for MACE (Table 3). After adjustment for conventional cardiovascular risk factors, including hemodialysis, a high ABI value was an independent predictor of MACE (HR, 2.07; 95% CI, 1.02–4.20; P = 0.044). Multivariate analysis also identified increased age, previous myocardial infarction, previous cerebral infarction, diabetes, hemodialysis, previous heart failure, and smoking as independent predictors of MACE.

**Table 3:** Univariate and multivariate Cox proportional hazard analyses for MACE.

	Univariate analysis		Multivariate analysis	
Variables	HR (95% CI)	P value	HR (95% CI)	P value
ABI > 1.4	2.52 (1.28–4.93)	0.007	2.07 (1.02–4.20)	0.044
Age (for each 1–year increase)	1.04 (1.03–1.06)	< 0.001	1.04 (1.03–1.06)	< 0.001
female	0.72 (0.50–1.02)	0.070		
BMI (for each 1–kg/m^2^ increase)	0.97 (0.93–1.01)	0.254		
Coronary heart disease	1.30 (0.89–1.90)	0.163		
Previous myocardial infarction	1.56 (1.07–2.27)	0.020	1.47 (1.00–2.16)	0.001
Previous cerebral infarction	2.19 (1.36–3.54)	0.001	1.93 (1.20–3.12)	0.007
Hypertension	1.12 (0.83–1.53)	0.440		
Dyslipidemia	0.74 (0.54–1.08)	0.058		
Diabetes	1.67 (1.22–2.29)	0.001	1.42 (1.03–1.96)	0.028
Atrial fibrillation	1.38 (0.92–2.09)	0.117		
Hemodialysis	3.41 (1.97–5.90)	< 0.001	3.03 (1.66–5.55)	< 0.001
Smoking habit	1.66 (1.22–2.26)	0.001	1.54 (1.13–2.10)	0.006
Hb (for each 0.1 g/L increase)	0.88 (0.81–0.95)	0.003	0.95 (0.87–1.04)	0.956
Previous heart failure	2.61 (1.69–4.02)	< 0.001	2.88 (1.86–4.45)	< 0.001

ABI, ankle brachial index; BMI, body mass index; CI, confidence interval; Hb, hemoglobin; HR, hazard ratio.

In addition, we performed univariate and multivariate Cox proportional analysis for all-cause and cardiovascular-associated death (S1 and S2 Tables). In multivariate analysis, high ABI was not an independent predictor for all-cause death (HR, 1.42; 95% CI, 0.78–2.57; P = 0.247) and cardiovascular-associated death (HR, 2.14; 95% CI, 0.94–4.86; P = 0.068).

### Predictors of MACE in patients without hemodialysis

In a separate analysis, we excluded patients on hemodialysis (n = 81) and included eGFR as a variable into the multivariable analysis model in order to assess the prognostic value of high ABI in patients without hemodialysis. A total of 5 MACE occurred in patients with high ABI (n = 52). In patients with normal ABI (n = 2286), a total of 153 MACE occurred. As shown in S3 Table, high ABI was not an independent predictor of MACE in this cohort.

## Discussion

In the present study, patients with high ABI exhibited significantly reduced BMI, hemoglobin and decreased eGFR, and more frequent chronic kidney disease (including hemodialysis) than those with normal ABI. In the multivariate logistic regression analysis, hemodialysis and lower BMI were independently associated with high ABI. Furthermore, the outcomes of patients with high ABI were evaluated, which demonstrated that the incidence of MACE, all-cause death, and cardiovascular-associated death were significantly higher in patients with high ABI values compared to those with normal ABI values. Moreover, multivariate Cox proportional hazard analysis adjusted for traditional cardiovascular risk factors, including hemodialysis, showed that a high ABI value was an independent predictor of MACE.

Few studies have evaluated the association of high ABI with prevalent diseases. Aboyans et al. reported a strong association between diabetes mellitus and high ABI [7]. Allison et al. also identified a positive association between diabetes mellitus and ABI values > 1.4 [16]. Inconsistent with these studies, in the ARIC study, patients with high ABI were not characterized by an atherosclerosis risk factor profile, including diabetes mellitus [8]. In the current study, no differences in traditional cardiovascular risk factors, including increased age, male sex, hypertension, diabetes mellitus, smoking, previous coronary heart disease, cerebral infarction, and heart failure, were detected in the baseline characteristics between the groups. Consistent with a previous study, dyslipidemia was inversely associated with high ABI [16]. The high ABI group exhibited significantly reduced BMI and hemoglobin, and frequent chronic renal dysfunction (including hemodialysis), as compared with the normal ABI group. Notably, multiple logistic regression analysis revealed that diabetes was not an independent predictor and hemodialysis was strongly associated with high ABI.

When the ankle artery is incompressible or the systolic pressure of the ankle artery is much higher than that of the brachial artery, the ABI value is abnormally high. This phenomenon is thought to occur in patients with diabetes mellitus and end-stage renal disease due to their non-compressible vessels caused by medial artery calcification [5, 6]. One possible explanation for the strong association of hemodialysis with high ABI in the present study can be the larger population of patients undergoing hemodialysis in Japan than other countries [17]. In order to determine whether hemodialysis is the dominant risk factor for high ABI, we have also performed Cox proportional hazard analysis in patients without a history of hemodialysis. In this cohort, high ABI was not an independent predictor of MACE. This result suggests that hemodialysis is an important confounder for high ABI. The other independent predictor for high ABI value was lower BMI. Recently, Kovacic et al. [18] showed that BMI was an independent inverse predictor of coronary artery calcification in 9,993 consecutive patients. Low BMI is known to be associated with decreased bone mineral density [19], which in turn is associated with increased vascular calcification, a relationship referred to as the ‘calcification paradox’ [20]. Bucay et al. [21] showed that osteoprotegerin played an important role in this pathway. In their experiment, osteoprotegerin-deficient mice exhibited a decrease in total bone density. Interestingly, osteoprotegerin-deficient mice also exhibited medial calcification of the aorta. Kovacic et al. [18] speculated that in this pathway, low BMI is associated with increased vascular calcification. Taken together, these findings suggest that the relationship between low BMI and high ABI is likely mediated via the effect of BMI on medial artery calcification.

In addition, regarding the prognosis of patients with high ABI, previous studies have reported inconsistent results. The SHS is a large-scale and long epidemiologic study in Native Americans, which consisted of 4,549 participants aged 45 to 74 years. In this study, 404 participants (9.2%) had high ABI (> 1.4) or incompressible arteries and this group had a higher risk of all-cause and cardiovascular associated death than the group with ABI between 0.9 to 1.4 [9]. The CHS is a community based longitudinal study of Medicare-eligible adults aged 65 years and older at enrollment conducted across four communities in the United States [10]. The investigators examined 5,748 participants for whom ABI was recorded at baseline, and identified 66 participants (1.1%) with high ABI (> 1.4). Unlike the results of the SHS, high ABI was not associated with fatal and nonfatal cardiovascular events after adjustment for confounders. Recently, Hendriks et al. evaluated 7,538 patients with a high risk for cardiovascular disease, and reported that high ABI was not associated with all-cause death, cardiovascular-associated death, or stroke [22]. Furthermore, few studies have reported the prognosis of Japanese patients with high ABI. Ono et al. indicated that patients with high ABI values had a poor prognosis for all-cause and cardiovascular-associated mortality [23]. Although they performed a large-scale prospective study, their cohort solely consisted of patients on hemodialysis.

In the present study, a high ABI was a parameter that independently predicted MACE. In addition to a high ABI value, increased age, previous myocardial infarction, heart failure and cerebral infarction, diabetes, hemodialysis, and smoking were independent predictors of MACE, which was consistent with previous studies [15, 24].

The pathophysiologic mechanism underlying the association between high ABI and poor prognosis has not been clearly elucidated. However, it is generally well accepted that a high ABI reflects increased arterial stiffness caused by medial artery calcification [5–8], and this mechanism likely contributes to the overall association. Vlachopoulos et al. [25] performed a meta-analysis of 17 longitudinal studies and found that arterial stiffness is an independent predictor of future cardiovascular events. Vascular stiffness is reported to contribute to left ventricular afterload, left ventricular hypertrophy, reduced coronary flow, carotid wall thickness and development of stenosis and plaques, which increase cardiovascular and cerebrovascular events [26]. This is one possible explanation for how high ABI independently increases the risk for MACE.

In our unadjusted analysis, high ABI was associated with all-cause and cardiovascular-associated death. However, high ABI was not associated with all-cause death after adjustment for cardiovascular risk factors (HR, 1.42; 95% CI, 0.78–2.57; P = 0.247). High ABI tended to be a risk factor for cardiovascular-associated death, although the HR did not reach statistical significance in the multivariate Cox proportional hazard analysis (HR, 2.14; 95% CI, 0.94–4.86; P = 0.068). In this study, we concluded that high ABI independently predicts MACE (primary endpoint), including cardiovascular-associated death, myocardial infarction, and stroke. As stated above, vascular stiffness is reported to contribute to cardiovascular and cerebrovascular events [26], and therefore the association of high ABI with future cardiovascular events might be stronger than that with the all-cause death, although this remains to be confirmed.

Our findings did not only indicate that patients receiving hemodialysis had poor outcomes, as multivariate Cox proportional hazard analysis adjusted for confounding variables (including hemodialysis) showed that high ABI was an independent predictor for MACE. These findings suggest that patients with high ABI values should be monitored, as the increased ABI values may be an indicator of increased risk for poor outcomes, even if ABI values are 1.0–1.4 in the other leg.

The current study included a large data set and was able to detect a sufficient number of cardiovascular events. However, this study has several potential limitations. First, the current study was a single center, retrospective analysis that consisted of patients who were hospitalized for cardiovascular disease. Therefore, our sample study group differed from the general population. Second, ABI was measured by an oscillometric method. When measuring ABI, the Doppler method is recommended, because of its increased reproducibility compared to the oscillometric method, which may have led to an overestimation of pressure [27]. Third, the number of patients in the high ABI group was small; thus, they may have limited statistical power. One potential reason for why high ABI was not associated with cardiovascular-associated death alone but with MACE might be small sample size of patients with high ABI. Therefore, a larger prospective study is required to confirm the current findings. Finally, we were not able to completely clarify the pathophysiologic mechanism underlying the relationship between high ABI and prognosis of cardiovascular events. Future investigation is warranted in order to determine the pathophysiologic mechanism whereby high ABI leads to poor prognosis.

## Conclusion

The current study showed that hemodialysis was the strongest independent predictor of high ABI. Patients with high ABI values exhibited a higher incidence of MACE, cardiovascular-associated death, and all-cause death as compared with those with normal ABI values. High ABI was an independent predictor of MACE. Notably, patients with ABI values > 1.4 exhibited a higher incidence of future cardiovascular events.

## Supporting Information

S1 Table Univariate and multivariate Cox proportional hazard analyses for all-cause death. (DOCX)

S2 Table Univariate and multivariate Cox proportional hazard analyses for cardiovascular-associated death. (DOCX)

S3 Table Univariate and multivariate Cox proportional hazard analyses for MACE after excluding hemodialysis patients. (DOCX)
