# Supplementary material for: Clinical Characteristics and Outcomes of Patients with High Ankle-Brachial Index from the IMPACT-ABI Study
Source: PLoS One. 2016 Nov 23;11(11):e0167150. doi: 10.1371/journal.pone.0167150 (PMC5120846; doi:10.1371/journal.pone.0167150)
Supplement: S1 Table — (DOCX) [file pone.0167150.s001.docx]

**S1 Table.**

|  | Univariate analysis | | Multivariate analysis | |
| --- | --- | --- | --- | --- |
| Variables | HR (95% CI) | P value | HR (95% CI) | P value |
| ABI > 1.4 | 1.90 (1.06–3.39) | 0.029 | 1.42 (0.78–2.57) | 0.247 |
| Age (for each 1–year increase) | 1.07 (1.05–1.08) | < 0.001 | 1.06 (1.05–1.07) | < 0.001 |
| female | 0.66 (0.50–0.87) | 0.003 | 0.54 (0.40–0.72) | < 0.001 |
| BMI (for each 1–kg/m^2^ increase) | 0.95 (0.90–0.96) | < 0.001 | 0.95 (0.92–0.99) | 0.011 |
| Coronary heart disease | 1.24 (0.92–1.65) | 0.144 |  |  |
| Previous myocardial infarction | 1.39 (1.03–1.86) | 0.028 | 1.41 (1.04–1.91) | 0.024 |
| Previous cerebral infarction | 1.80 (1.22–2.65) | 0.003 | 1.64 (1.11–2.44) | 0.015 |
| Hypertension | 1.11 (0.88–1.40) | 0.357 |  |  |
| Dyslipidemia | 0.60 (0.47–0.77) | < 0.001 | 0.67 (0.52–0.86) | 0.002 |
| Diabetes | 1.29 (1.01–1.65) | 0.041 | 1.19 (0.92–1.54) | 0.164 |
| Atrial fibrillation | 1.12 (0.80–1.57) | 0.495 |  |  |
| Hemodialysis | 2.85 (1.83–4.45) | < 0.001 | 2.09 (1.30–3.38) | 0.002 |
| Smoking habit | 1.22 (0.97–1.52) | 0.083 |  |  |
| Hb (for each 0.1 g/L increase) | 0.78 (0.73–0.82) | < 0.001 | 0.84 (0.78–0.90) | < 0.001 |
| Previous heart failure | 2.34 (1.67–3.30) | < 0.001 | 2.54 (1.80–3.58) | < 0.001 |

ABI, ankle brachial index; BMI, body mass index; CI, confidence interval; Hb, hemoglobin; HR, hazard ratio.
