# Supplementary material for: Clinical Characteristics and Outcomes of Patients with High Ankle-Brachial Index from the IMPACT-ABI Study
Source: PLoS One. 2016 Nov 23;11(11):e0167150. doi: 10.1371/journal.pone.0167150 (PMC5120846; doi:10.1371/journal.pone.0167150)
Supplement: S2 Table — (DOCX) [file pone.0167150.s002.docx]

**S2 Table.**

|  | Univariate analysis | | Multivariate analysis | |
| --- | --- | --- | --- | --- |
| Variables | HR (95% CI) | P value | HR (95% CI) | P value |
| ABI > 1.4 | 2.96 (1.37–6.36) | 0.005 | 2.14 (0.94–4.86) | 0.068 |
| Age (for each 1–year increase) | 1.05 (1.03–1.07) | < 0.001 | 1.05 (1.03–1.07) | < 0.001 |
| female | 0.75 (0.49–1.14) | 0.187 |  |  |
| BMI (for each 1–kg/m^2^ increase) | 0.96 (0.92–1.01) | 0.203 |  |  |
| Coronary heart disease | 1.31 (0.83–2.07) | 0.240 |  |  |
| Previous myocardial infarction | 1.77 (1.14–2.76) | 0.011 | 1.95 (1.23–3.09) | 0.004 |
| Previous cerebral infarction | 2.44 (1.39–4.27) | 0.002 | 2.22 (1.27–3.91) | 0.005 |
| Hypertension | 0.95 (0.65–1.37) | 0.788 |  |  |
| Dyslipidemia | 0.54 (0.36–0.80) | 0.003 | 0.49 (0.32–0.74) | 0.001 |
| Diabetes | 1.49 (1.01–2.20) | 0.042 | 1.34 (0.89–1.99) | 0.151 |
| Atrial fibrillation | 1.87 (1.18–2.96) | 0.007 | 1.43 (0.88–2.32) | 0.145 |
| Hemodialysis | 4.01 (2.15–7.49) | < 0.001 | 3.14 (1.55–6.36) | 0.001 |
| Smoking habit | 1.50 (1.04–2.16) | 0.003 | 1.65 (1.13–2.41) | 0.009 |
| Hb (for each 0.1 g/L increase) | 0.84 (0.76–0.93) | 0.001 | 0.93 (0.84–1.03) | 0.210 |
| Previous heart failure | 3.83 (2.40–6.11) | < 0.001 | 3.83 (2.34–6.27) | < 0.001 |

ABI, ankle brachial index; BMI, body mass index; CI, confidence interval; Hb, hemoglobin; HR, hazard ratio.
