# Supplementary material for: Clinical Characteristics and Outcomes of Patients with High Ankle-Brachial Index from the IMPACT-ABI Study
Source: PLoS One. 2016 Nov 23;11(11):e0167150. doi: 10.1371/journal.pone.0167150 (PMC5120846; doi:10.1371/journal.pone.0167150)
Supplement: S3 Table — (DOCX) [file pone.0167150.s003.docx]

**S3 Table.**

|  | Univariate analysis | | Multivariate analysis | |
| --- | --- | --- | --- | --- |
| Variables | HR (95% CI) | P value | HR (95% CI) | P value |
| ABI > 1.4 | 1.77 (0.72–4.32) | 0.207 |  |  |
| Age (for each 1–year increase) | 1.64 (1.40–1.93) | < 0.001 | 1.57 (1.32–1.87) | < 0.001 |
| female | 0.73 (0.50–1.05) | 0.095 |  |  |
| BMI (for each 1–kg/m^2^ increase) | 0.56 (0.94–1.03) | 0.254 |  |  |
| Coronary heart disease | 1.22 (0.81–1.82) | 0.332 |  |  |
| Previous myocardial infarction | 1.63 (1.11–2.39) | 0.012 | 1.43 (0.97–2.12) | 0.070 |
| Previous cerebral infarction | 2.13 (1.29–3.53) | 0.003 | 1.71 (1.03–2.84) | 0.037 |
| Hypertension | 1.11 (0.81–1.53) | 0.501 |  |  |
| Dyslipidemia | 0.79 (0.54–1.08) | 0.155 |  |  |
| Diabetes | 1.53 (1.09–2.13) | 0.012 | 1.29 (0.92–1.81) | 0.14 |
| Atrial fibrillation | 1.46 (0.96–2.23) | 0.075 |  |  |
| eGFR | 0.97 (0.96–0.98) | < 0.001 | 0.98 (0.97–0.99) | 0.002 |
| Smoking habit | 1.37 (1.00–1.88) | 0.046 | 1.38 (1.00–1.90) | 0.048 |
| Hb (for each 0.1 g/L increase) | 0.91 (0.84–1.00) | 0.057 |  |  |
| Previous heart failure | 2.74 (1.76–4.27) | < 0.001 | 2.67 (1.70–4.20) | < 0.001 |

ABI, ankle brachial index; BMI, body mass index; CI, confidence interval; eGFR, estimated glomerular filtration rate; Hb, hemoglobin; HR, hazard ratio.
